# Avicularin Attenuates Lead-Induced Impairment of Hepatic Glucose Metabolism by Inhibiting the ER Stress-Mediated Inflammatory Pathway

**DOI:** 10.3390/nu14224806

**Published:** 2022-11-13

**Authors:** Ting Qiu, Jia-Xue Shi, Chao Cheng, Hong Jiang, Hai-Nan Ruan, Jun Li, Chan-Min Liu

**Affiliations:** School of Life Science, Jiangsu Normal University, No. 101, Shanghai Road, Tongshan New Area, Xuzhou 221116, China

**Keywords:** avicularin, lead, glucose metabolism, inflammation, endoplasmic reticulum stress, liver damage

## Abstract

Lead (Pb), an environmental hazard, causes several human diseases. Avicularin (Avi), a main dietary flavonoid found in several plants and fruits, exhibits potential protective properties on organs. However, the molecular mechanisms of Avi’s protective effects against Pb-induced damage are not clear. In our study, the effects of Avi on Pb-induced hepatotoxicity were evaluated using ICR mice. We have revealed for the first time that treatment with Avi significantly reduced hepatic inflammation, endoplasmic reticulum stress (ERS) and glucose metabolism disorder induced by Pb. Avi decreased the serum biochemical indicators of glucose metabolism. Avi increased the activities of glycogenolysis rate-limiting enzyme hexokinase (HK), pyruvate kinase (PK), glucokinase (GK) and glycogen phosphorylase (PYG) and inhibited the activities of gluconeogenesis rate-limiting enzyme phosphoenolpyruvate carboxy kinase (PEPCK) and glucose-6-phosphate dehydrogenase (G6PD). Avi decreased the protein expression levels of glucose-regulated protein 78 (GRP78), phosphorylated inositol requiring enzyme 1 (p-IRE1), phosphorylated RNA-dependent protein kinase-like ER kinase (p-PERK) and phosphorylated eukaryotic initiation factor 2α (p-eIF2α). The levels of tumor necrosis factor-alpha (TNF-α) and interleukin-6 (IL-6) were decreased in the liver as a result of Avi suppression Pb-induced inflammation. These results indicated that Avi attenuated Pb-induced impairment of hepatic glucose metabolism by the ERS and inflammation pathway.

## 1. Introduction

Lead (Pb), an environmental hazard, causes severe diseases in the liver, kidney, cardiovascular system, hematopoietic system, reproductive systems and nervous system [1,2]. Pb can be obtained from the intake of food and drinking water [3,4,5]. It was reported that Pb exposure is closely associated with coronary artery disease [6]. Pb exposure can interfere with glucose metabolism and promotes diabetes in animals [2,3]. Pb exposure can impair brain glucose metabolism by affecting the expression levels of the key regulatory enzyme [7,8]. Pb exposure has also caused insulin resistance and metabolism disorder in the livers of experimental animals by regulating the activity of glycogenolysis and gluconeogenesis enzymes, including hexokinase (HK), pyruvate kinase (PK), glucokinase (GK), phosphoenolpyruvate carboxy kinase (PEPCK) and glucose-6-phosphate dehydrogenase (G6PD) [3,9,10]. Pb exposure also induces liver damage though the endoplasmic reticulum stress (ERS) pathway, which may cause glucose metabolism disorder [11,12]. However, the mechanisms of Pb-induced glucose metabolism disorder are still unclear.

**Figure 1:**
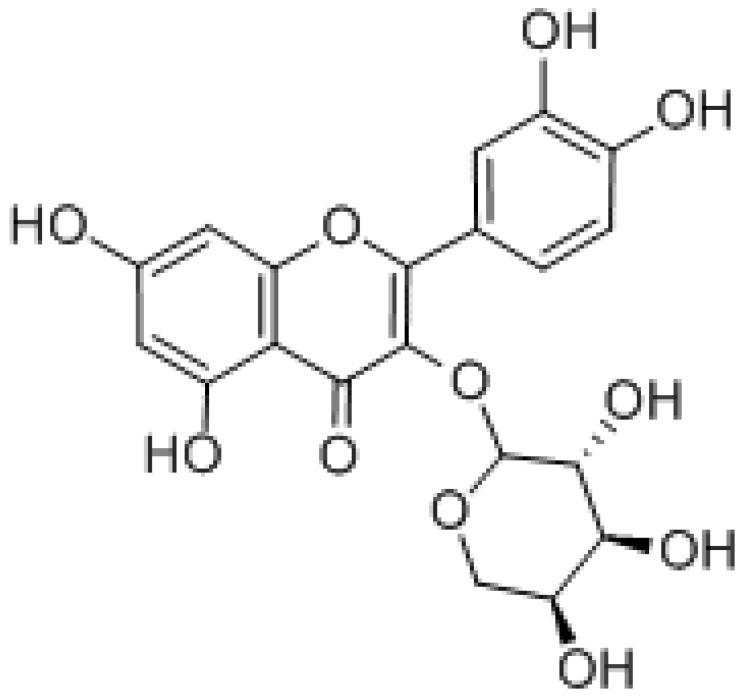
Structure of avicularin.

Avicularin (Avi, quercetin-3-alpha-L-arabinofuranoside) (Figure 1) is a dietary flavonoid found in several plants and fruits, which has displayed multiple pharmacological effects, including anti-tumor, anti-oxidative, anti-inflammatory, anti-depressant and hepatoprotective properties [13,14,15]. Avi supplementation improved insulin resistance by regulating the expression levels of glycogen phosphorylase (PYG), PEPCK, PK and glucose-6-phosphatase (G6PC) [13,14,16]. Avi diminished liver inflammation by inhibiting the expression of proinflammatory factors including tumor necrosis factor (TNF) α, interleukin 1β (IL-1β) [15,17]. More recently, Avi supplementation exerted a neuroprotective effect by inhibiting Alzheimer’s disease in rats [18]. However, it is unknown whether Avi can improve Pb-induced hepatic ERS and glucose metabolism disorder.

Therefore, Avi was evaluated for the first time for its hepatoprotective effects against Pb-induced hepatic inflammation and glucose metabolism disorder, and this study further clarified the role of the ERS pathway in Avi protection.

## 2. Materials and Methods

### 2.1. Chemicals and Reagents

Avicularin (purity > 99%) and lead acetate were purchased from Sigma Chemical Co. (St. Louis, MO, USA). The anti-p-IRE1, anti-PERK, anti-p-eIF2α, anti-GRP78, anti-PYG, anti-GK, anti-PEPCK, anti-G6PC, anti-TNF-α, anti-IL-1β and anti-β-actin antibodies were provided by Abcam (Cambridge, MA, USA and Santa Cruz Biotechnology, CA, USA) [5].

### 2.2. Animals and Ethics

Fifty male ICR mice (20 ± 1 g) were obtained from Beijing HFK Bioscience Co., Ltd, (Beijing, China). All experiment processes were approved by Jiangsu Normal University Committees (approval No. 3/5/2018) and performed according to the relevant guidelines.

### 2.3. Experimental Design

The mice were positioned in the animal room, with a temperature of 23 ± 1 °C, a 12 h dark/light cycle and relative humidity (55 ± 5)%. After 1 week of adaptive rearing, the mice were randomly divided into five groups (10 mice/group): (1) Normal control group; (2) Pb group; (3) Pb + Avi (25 mg/kg) group; (4) Pb + Avi (50 mg/kg) group; (5) Avi (50 mg/kg) control group. In group (1), the mice received deionized water as drinking water. In groups (2), (3) and (4), the procedure of Pb (0.05% Pb) inducing glucose metabolism was performed as described previously in the literature [3,11]. In groups (3), (4) and (5), the mice were also supplied with Avi 25 or 50 mg/kg, intragastrically once daily. The dose of Avi selected in this study was based on previously published data on the protective effect of Avi [13,18].

At the end of 3 months, blood and liver samples were collected immediately after the mice were decapitated. All the samples were frozen at −80 °C until they were assayed.

### 2.4. Biochemical Analysis

At the end of 3 months, an oral glucose tolerance test (OGTT) was conducted. The hepatic GK activity was measured using commercial kits from Westang Biotechnology company (Shanghai, China) [5,13,19]. The activities of serum alanine transaminase (ALT) (#C009-3-1), aspartate transaminase (AST) (# C010-3-1), glucose (Glu) (#F006-1-1), insulin (IS) (#H203-1-1), hepatic glycogen (A043-1-1), PEPCK (#A131-1-1), HK (#A077-3-1) and G6PD (#M015) were measured using commercial kits from Jiancheng Institute of Biotechnology (Nanjing, China) [5,13].

### 2.5. Western Blotting Analysis

The expression levels of the p-IRE1, p-PERK, p-eIF2α, GRP78, PYG, GK, PEPCK, G6PC, TNF-α, IL-1β and β-actin were analyzed by Western blot analysis according to the manufacturer’s guidelines (Bio/Rad, Hercules, CA, USA). Image J 1.42 software (NIH Bethesda, MD, USA) was used to quantitate band intensities [5].

### 2.6. Statistical Analysis

The data was presented as mean ± standard error (SE). For multiple comparisons after the one-way variance (ANOVA) test, Tukey’s test was applied.

## 3. Results

### 3.1. Avi Rescues Pb-Induced Liver Dysfunction

Pb exposure resulted in a significant increase in serum ALT and AST activities compared to the control group (25.21 ± 1.07 and 37.42 ± 2.51) by 243.59% and 163.01%, respectively. The treatment with Avi (25 and 50 mg/kg) reduced the activities of ALT (by 15.61% and 34.09%, respectively) and AST (by 19.71% and 27.29%, respectively) compared to the Pb group (86.62 ± 1.76 and 98.42 ± 4.50), showing a dose-dependent relationship (R^2^_ALT_ = 0.9976, R^2^_AST_ = 0.9382) (Table 1). Treatment with Avi only had no significant effect on the activities of these enzymes.

### 3.2. Effects of Avi on Pb-Induced Insulin Resistance

In order to examine the therapeutic potential of Avi on Pb-induced insulin resistance, serum FBG and IS levels were determined. Pb exposure increased the levels of serum FBG, IS and HOMA-IS compared to the normal control group by 30.50%, 153.03% and 218.18%, respectively. Avi (25 and 50 mg/kg) treatment decreased the serum FBG level (by 13.37% and 22.32%, respectively), IS (by 21.56% and 35.33%, respectively) and HOMA-IS (by 22.95% and 31.43%, respectively) compared to the Pb group (Table 1). Therefore, Avi only had no significant effect on the levels of serum FBG, IS and HOMA-IS.

### 3.3. Effects of Avi on the Abnormal Activities of Pb-Induced Glucose Metabolism in the Liver

To evaluate the effect of Avi on the glucose metabolism activities in the liver, we measure the activities of GK, PK, HK, PEPCK and G6PD in the liver. Pb exposure decreased the activities of the glycogenolysis enzymes GK, PK and HK compared to the normal control group by 65.75%, 43.16% and 63.55%, respectively. Pb exposure increased the activities of the gluconeogenesis enzymes PEPCK and G6PD compared to the normal control group by 96.23% and 184.75%, respectively. In contrast, Avi (25 and 50 mg/kg) treatment restored the activities of those glucose metabolism enzymes (Table 2). Therefore, Avi only had no significant effect on those enzyme activities in the liver.

### 3.4. Avi Regulated the Expression Levels of Glucose Metabolism Enzymes in the Liver

**Figure 2:**
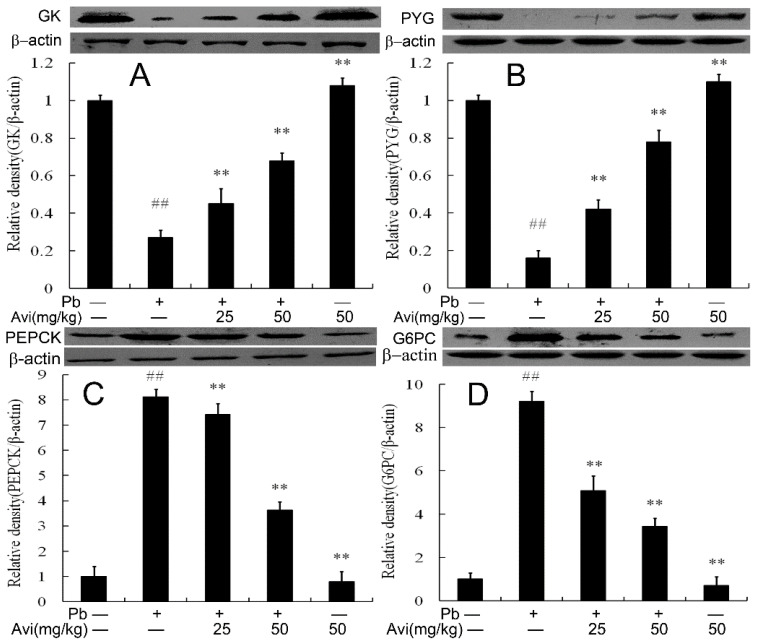
The protein expression of glucose metabolism in the livers of mice. (**A**) Relative density analysis of the GK protein bands; (**B**) Relative density analysis of the PYG protein bands; (**C**) Relative density analysis of the PEPCK protein bands; (**D**) Relative density analysis of the G6PC protein bands. β-actin was probed as an internal control in the relative density analysis. The vehicle control is set as 1.0. Data are expressed as mean ± S.E.M. and representative of at least three independent experiments (*n* = 3, individual animals). ## *p* < 0.05 compared with the control group; ** *p* < 0.05, vs., Pb-treated group.

Western blotting was used to determine liver glucose metabolism enzyme expression. As depicted in Figure 1, Pb exposure decreased the protein expression levels of GK and PYG and increased the levels of PEPCK and G6PC compared with the normal control group. However, Avi (25 and 50 mg/kg) restored the protein expression levels of those glucose metabolism enzymes (Figure 2).

### 3.5. Avi Suppressed Hepatic Inflammation

**Figure 3:**
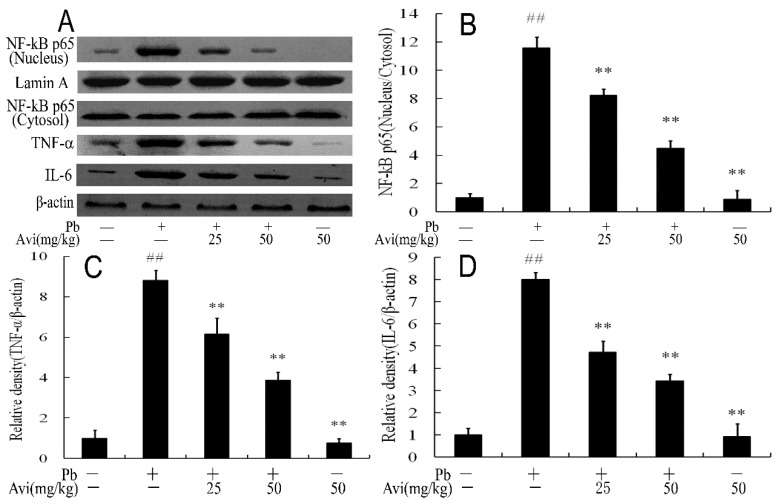
Avicularin (Avi) inhibited Pb-induced inflammation in the livers of mice. (**A**) Western blot analysis of the NF-κB p65, TNF-α and IL-6 proteins in the livers; (**B**) Relative density analysis of the NF-κB p65 bands; (**C**) Relative density analysis of the TNF-α protein bands; (**D**) Relative density analysis of the IL-6 protein bands. β-actin and Lamin A were probed as the internal control in the relative density analysis. The vehicle control is set as 1.0. Data are expressed as mean ± S.E.M. and representative of at least three independent experiments (*n* = 3, individual animals). ## *p* < 0.05 compared with the control group; ** *p* < 0.05, vs., Pb-treated group.

Inflammation plays an important role in the pathogenesis of fatty liver disease. We evaluated the NF-κB nuclear translocation and the expression of pro-inflammatory cytokines TNF-α and IL-6 in hepatic tissue. As displayed in Figure 3, Pb exposure increased the levels of TNF-α, IL-6 and the NF-κB nuclear transcriptional activity compared to the normal control group. Avi (25 and 50 mg/kg) treatment decreased the expression of the inflammatory factor compared to the Pb group (*p* < 0.05).

### 3.6. Avi Suppresses the ERS Pathway in the Liver

**Figure 4:**
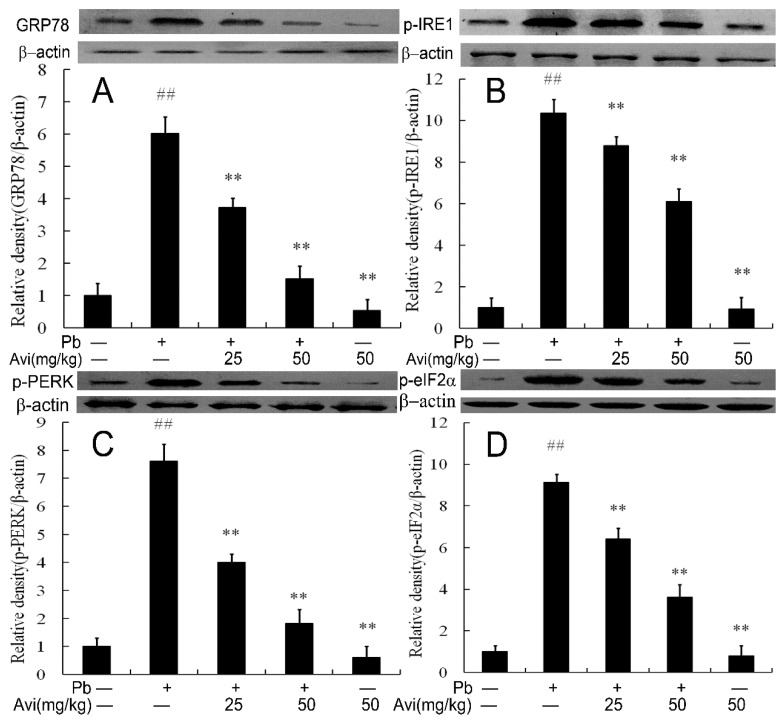
Avicularin (Avi) inhibited the ERS pathway in the livers of mice. (**A**) Western blot analysis of the GRP78 proteins in the livers; (**B**) Relative density analysis of the p-IRE1 bands; (**C**) Relative density analysis of the p-PERK protein bands; (**D**) Relative density analysis of the p-eIF2α protein bands. β-actin was probed as the internal control in the relative density analysis. The vehicle control is set as 1.0. Data are expressed as mean ± S.E.M. and representative of at least three independent experiments (individual animals). ## *p* < 0.05 compared with the control group; ** *p* < 0.05, vs., Pb-treated group.

ERS is involved in inflammation and glucose metabolism. We further examined the expression levels of GRP78, p-IRE1, p-PERK and p-eIF2α. As depicted in Figure 4, the expression levels of GRP78, p-IRE1, p-PERK and p-eIF2α were up-regulated in the Pb group. In contrast to the Pb group, treatment with different dosages of Avi significantly decreased the phosphorylation levels of these proteins.

## 4. Discussion

Pb is a ubiquitous, persistent and non-essential toxic heavy metal which can induce the disorder of glucose metabolism [2,3]. Pb exposure induces multiple liver injuries [9,10,11]. Our study found that Pb induced hepatic inflammation, ERS and glucose metabolism disorder (Table 1). Interestingly, we found that Avi supplementation mitigated Pb-induced liver injury.

Pb exposure could cause hyperglycemia and insulin resistance in many organs [3,5,19]. Pb exposure can interfere with glucose metabolism and promotes diabetes in animals [3,5]. Current research shows that Pb exposure increases the levels of serum FBG, IS and HOMA-IS compared with normal control groups, which indicates hyperglycemia [2,7]. Avi treatment showed a hypoglycemic effect in a diabetes model [13,16]. Our results revealed that Avi supplementation inhibited Pb-induced hyperglycemia and insulin resistance in mice (Table 1).

PYG is the rate-limiting enzyme in glycogenolysis [13,20]. PK and HK are the key glycolytic enzymes that control glycolysis rate, which are the key factors that administer glucose production [8,20]. GK is the rate-limiting enzyme modulating glucose metabolism, glycogen synthesis and insulin secretion [16,21]. PEPCK is a rate-limiting enzyme in gluconeogenesis, which also influences blood glucose levels and hepatic glucose production [3,10,13]. Research has shown that Pb decreased the activities of HK, PK and increased the activities of PEPCK, G6PC in brain and liver tissue [3,8]. Additionally, the expression of HK, PK, GK and PYG was significantly decreased [7,9] and the expression of PEPCK, G6PC was increased in the Pb group compared with controls [3,10]. Research found that Avi could up-regulate the expression of PK and PYG to alleviate glucose metabolism disorder [13]. In our current work, we found that Avi treatment recovered the activations of HK, PK, GK, PYG, PEPCK and G6PC in the Pb group (Figure 2). Thus, the above results indicate that Avi ameliorated Pb-induced glucose metabolism disorder.

Inflammatory response is significantly associated with glucose metabolism [22]. Pb exposure reportedly stimulated inflammation and abnormal metabolism of gluconeogenesis and glycogenolysis in the liver [3]. Several studies revealed that Avi could inhibit the formation of inflammasome in the liver and brain of different experimental models, thus reducing toxin-induced tissue damage [15,17,23]. Avi could inhibit inflammation and fibrosis in osteoarthritis [24]. Avi could also prevent the release of IL-1β, IL-6 and TNF-α in various cells [24,25,26]. The results of this experiment showed that Pb stimulates NF-κB nuclear transcriptional activity and the release of IL-1β and TNF-α. Meanwhile, Avi could significantly prevent the secretion of these pro-inflammatory cytokines (Figure 3), indicating that Avi alleviates liver injury by inhibiting Pb-induced inflammation.

ERS is known as the “unfolded protein response (UPR),” which has three classical signaling pathways, IRE1α, PERK and ATF6 [5,27]. Excess ERS will not only induce apoptosis and inflammatory response but will also cause glucose metabolism disorder and other physiological diseases [27,28,29]. Hepatic ERS can induce gluconeogenesis by stimulating the activation of IRE1, PERK and eIF2α [30,31]. ERS disrupts insulin signaling and promotes hepatic insulin resistance and glucose production [31,32,33]. Pb was found to induce ERS in multiple tissues and cells, which further activated NF-κB and stimulated inflammation by the PERK and IRE1α signaling pathways [3,5,11]. As mentioned above, we found that Pb increased the expression of GRP78, p-IRE1, p-PERK and p-eIF2α, which indicated that Pb caused ERS in the liver [3]. However, Avi supplementation reduced the expression of GRP78, p-IRE1, p-PERK and p-eIF2α, and thus decreased Pb-induced ERS in the liver. We could claim that Avi was able to mitigate Pb-induced liver injury by inhibiting ERS (Figure 4).

**Figure 5:**
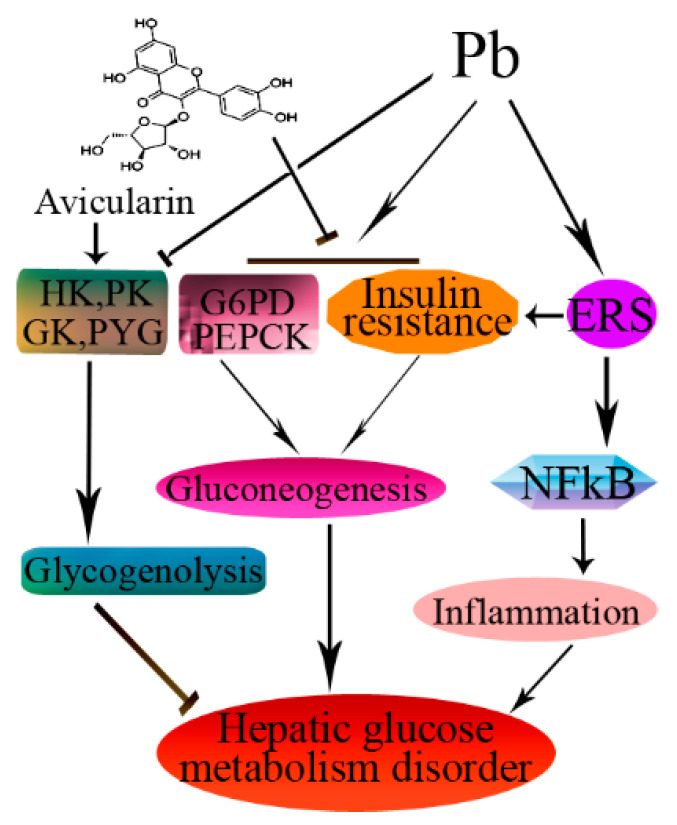
Schematic diagram showing the possible protective effects of Avicularin (Avi) in Pb-induced liver injury. The → indicates activation or induction, and ┤ indicates inhibition or blockade.

In conclusion, Avi significantly alleviated Pb-induced hepatic inflammation and glucose metabolism disorder and inflammation by inhibiting the ERS pathway (Figure 5). The hepatoprotection of Avi warrants further investigation in our future research.

## Figures and Tables

**Table 1:** Effect of avicularin on the serum biochemical parameters of mice.

	ALT (U/L)	AST (U/L)	FBG (mM/L)	IS (mM/L)	HOMA-IR
Control	25.21 ± 1.07	37.42 ± 2.51	3.64 ± 0.04	0.66 ± 0.02	0.11 ± 0.01
Pb	86.62 ± 1.76 #	98.42 ± 4.50 #	4.75 ± 0.08 #	1.67 ± 0.04 #	0.35 ± 0.01 #
Pb + Avi (25mg/kg)	73.10 ± 1.72 *	79.02 ± 2.17 *	4.02 ± 0.07 *	1.31 ± 0.03 *	0.24 ± 0.01 *
Pb + Avi (50mg/kg)	57.09 ± 1.67 *	71.56 ± 2.87 *	3.69 ± 0.04 *	1.08 ± 0.02 *	0.18 ± 0.01 *
Avi (50mg/kg)	43.04 ± 1.75 *	42.94 ± 4.16 *	3.63 ± 0.03 *	0.66 ± 0.03 *	0.11 ± 0.02 *

Data are expressed as mean ± S.E.M. (*n* = 6). One-way ANOVA was used for comparisons of multiple group means followed by post hoc testing. # *p* < 0.05, compared with the control group; * *p* < 0.05, vs., the Pb group.

**Table 2:** Effect of avicularin on the activities of those glucose metabolism enzymes in the liver.

	GK (U/g.prot)	HK (U/g.prot)	PK (U/g.prot)	PEPCK (U/g.prot)	D6PD (U/g.prot)
Control	1.81 ± 0.04	231.21 ± 11.74	170.85 ± 2.37	0.53 ± 0.04	0.59 ± 0.04
Pb	0.62 ± 0.01 #	131.41 ± 13.23 #	62.28 ± 4.96 #	1.04 ± 0.02 #	1.68 ± 0.06 #
Pb + Avi (25mg/kg)	0.88 ± 0.03 *	168.11 ± 5.94 *	119.84 ± 8.53 *	0.84 ± 0.02 *	1.31 ± 0.03 *
Pb + Avi (50mg/kg)	1.06 ± 0.02 *	188.22 ± 9.31 *	140.80 ± 5.76 *	0.72 ± 0.03 *	1.06 ± 0.05 *
Avi (50mg/kg)	1.82 ± 0.02 *	206.12 ± 5.94 *	163.93 ± 13.60 *	0.56 ± 0.02 *	0.73 ± 0.03 *

Data are expressed as mean ± S.E.M. (*n* = 6). One-way ANOVA was used for comparisons of multiple group means followed by post hoc testing. # *p* < 0.05, compared with the control group; * *p* < 0.05, vs., the Pb group.

## Abbreviation

ALT, alanine transaminase; ATF6, activating transcription factor 6; AST, aspartate transaminase; ERS, endoplasmic reticulum stress; eIF2α, eukaryotic initiation factor 2α; GK, glucokinase; G6PD, glucose-6-phosphate dehydrogenase; GRP78, glucose-regulated protein 78; IRE1, inositol requiring enzyme 1; HK, hexokinase; PEPCK, phosphoenolpyruvate carboxy kinase; PERK, RNA-dependent protein kinase-like ER kinase; PK, pyruvate kinase; PYG, glycogen phosphorylase; TC, total cholesterol; TG, triglycerides; TNF-α, tumor necrosis factor-alpha; IL-6, interleukin-6; NF-κB, nuclear factor-κB.
